# Genetic family studies and prospective evaluation for multisystem involvement are needed in LHON patients

**DOI:** 10.22336/rjo.2024.62

**Published:** 2024

**Authors:** Josef Finsterer

**Affiliations:** Neurology & Neurophysiology Center, Vienna, Austria

**Keywords:** mtDNA, LHON, visual acuity, idebenone, mitochondrial disorder

## Commentary

We read with interest the article by Iorga et al. about a 28-year-old man with Leber’s hereditary optic neuropathy (LHON) due to the variant m.11778G > A in *ND6* without reporting heteroplasmy rates [1]. The patient clinically presented with a subacute decrease in visual acuity in his right eye, followed one month later by a sustained visual acuity decrease in the left eye [1]. SD-OCT showed swollen optic nerve, pRNFL thickening of the upper, nasal, and lower quadrants of the right eye and the lower quadrant of the left eye, and thinning of the retinal ganglion cell layer [1]. MRI of the optic nerve and brain confirmed nerve swelling and showed brain atrophy [1]. The patient benefited 10 weeks after starting treatment with idebenone [1]. The study is impressive, but some points require further discussion.

The first point is that we disagree with the statement in the abstract that LHON is the most common maternally inherited mitochondrial disorder (MID) [1]. The most common maternally inherited MID is mitochondrial encephalopathy, lactic acidosis, and stroke-like episode (MELAS) syndrome, which is due to the variant m, 3243A > G in *MT-TL1* in 85% of cases. The prevalence of MELAS has been calculated to be 0.6 to 16/100000 [2,3].

The second point is that there is no discussion about LHON plus. LHON can manifest not only in the retinal arteries, retinal ganglion cells, and the optic nerve but also in many other organs, particularly in the brain [4] or myocardium [5]. Cerebral manifestations of LHON may include Harding’s disease, dystonia, or epilepsy. Cardiac manifestations of LHON may include hypertrophic or dilated cardiomyopathy.

The third point is that magnetic resonance imaging (MRI) results described as “symmetrical enlargement if intergyral grooves” indicate cerebral atrophy. Therefore, we should know whether diffuse cortical cerebral atrophy existed and whether the index patient had clinical manifestations of diffuse atrophy, such as cognitive impairment, motor deficits, or sensory impairment.

The fourth point is that it is incomprehensible why the neurological clinical examination was classified as normal. Since the patient had severe visual impairment, this should also have been recognized during the neurological examination. In addition, it is conceivable that cerebral atrophy is associated with mild cognitive impairment. Did the patient exhibit neuropsychological cognitive, motor, or sensory deficits during the clinical neurological examination?

The fifth point is that we disagree with the statement in **Table 1** that there is no spontaneous recovery in LHON [1]. On the contrary, there are numerous reports of LHON patients, in whom visual impairment spontaneously resolved [6]. This was most commonly described in LHON patients carrying the m.3460G > A variant.

The sixth point is that no long-term follow-up was conducted to assess whether idebenone was effective in the long term or only in the acute phase. In addition, how can one be sure that stabilization of visual acuity after 10 weeks of idebenone was due to the medication and not the natural progression of the disease?

The results of the fluorescence angiography are missing. Were there typical telangiectasias without leakage?

In summary, the interesting study has limitations that put the results and their interpretation into perspective. Removing these limitations could strengthen the conclusions and support the study’s message. Before the stabilization of visual acuity in LHON is attributed to idebenone, the spontaneous progression of the disease should be considered responsible. LHON patients should be prospectively evaluated for multisystem involvement. To assess whether the causative variant occurred sporadically or was inherited, genetic testing of first-degree relatives is mandatory.
